# Active protein quality control in quiescence: involvement of proteasomes, autophagy, and nucleus-vacuole junctions

**DOI:** 10.1080/27694127.2025.2507266

**Published:** 2025-05-31

**Authors:** Mihaela Pravica, Dina Franić, Mirta Boban

**Affiliations:** Croatian Institute for Brain Research, University of Zagreb School of Medicine, Zagreb, Croatia

**Keywords:** Nucleus-vacuole junction, proteasome storage granule, protein aggregate, protein quality control, quiescence, Vac8, yeast

## Abstract

Quiescence is a conserved, reversible state of proliferative arrest, characterized by changes in cell physiology and metabolism. Many cells spend a considerable part of their lifetime in quiescence, including adult stem cells or microorganisms facing unfavorable environmental conditions. Cells can remain quiescent for long periods of time while retaining their viability and reproductive capacity, indicating a need to maintain protein homeostasis. Given the changes in intracellular organization, it has been unclear how protein quality control (PQC) functions in quiescent cells. In our recent study, we examined model misfolded proteins expressed in glucose-depleted quiescent yeast cells and found that quiescent cells maintain an active PQC that relies primarily on selective protein degradation, requiring the ubiquitin-proteasome system, intact nucleus-vacuole junctions and autophagy. Our results highlight the relevance of mitigating misfolded proteins in quiescence.

Protein homeostasis is maintained by the protein quality control (PQC) system, a network of evolutionarily conserved pathways that mitigate the accumulation of misfolded proteins through protein refolding, selective protein degradation, and spatial sequestration to quality control compartments or deposits. Unlike proliferating cells, quiescent cells are unable to clear protein aggregates by retaining them in the mother cells during asymmetric cell divisions. In addition, glucose-depleted quiescent yeast cells induce autophagy and relocalize a large pool of the proteasomes from the nucleus to the nuclear periphery and cytoplasmic proteasome storage granules, structures that contain disassembled, inactive proteasomes. Thus, given the changes in the metabolism and intracellular organization, the operation of PQC in quiescent cells remains largely unclear.

In our recent study [1], we investigated PQC pathways in the quiescent cells of yeast *Saccharomyces cerevisiae*. We examined two model misfolded proteins, truncated Gnd1 (tGnd1) and small truncated Gnd1 (stGnd1), C-terminal truncation mutants of the 6-phosphogluconate dehydrogenase Gnd1, which are known substrates of the ubiquitin-proteasome system (UPS) in exponentially growing cells. We found that tGnd1 and stGnd1 were targeted for selective degradation, indicating that quiescent cells maintain an active degradation-mediated PQC. Degradation of stGnd1 and tGnd1 in quiescent cells required the E3 ubiquitin ligases San1 and Ubr1, and the deubiquitinase Rpn11, which is critical for the 26S proteasome function. These data suggest that quiescent cells retain a significant pool of fully assembled proteasomes that are engaged in the degradation of poly-ubiquitinated quality control substrates. Since protein synthesis is downregulated in quiescence, and newly synthesized proteins are the main source of protein misfolding, even moderate levels of assembled 26S proteasomes are likely sufficient to sustain degradation-mediated PQC in quiescent cells.

Given the upregulation of autophagy and nucleus-vacuole junctions (NVJ) in glucose-depleted yeast cells, we investigated their involvement in PQC during quiescence. NVJs are membrane contact sites between the nucleus and the vacuole, the yeast counterpart of the mammalian lysosome, which are formed by direct interaction between the outer nuclear membrane protein Nvj1 and the vacuolar membrane protein Vac8. We found that the misfolded truncated mutant tGnd1 was stabilized in quiescent cells of autophagy-deficient *atg1△* (*autophagy-related 1△*) or *atg8△* mutant, as well as in strain in which NVJs are disrupted by the *nvj1△* and *vac8△* deletion. In contrast to tGnd1, stGnd1 was not stabilized in autophagy or NVJ mutants, indicating that the involvement of the PQC pathways is substrate-specific. Inactivation of Cue5, the only known ubiquitin-binding selective autophagy receptor in yeast, had no effect on tGnd1 stability. The selective autophagy receptors Atg36, Atg39 and Atg40, previously implicated in Cue5-independent inclusion body autophagy, represent potential mediators of this process in quiescent cells, but they remain to be investigated. Consistent with the possibility that the involvement of NVJ and autophagy requires the localization of misfolded proteins to inclusions, we observed that tGnd1 formed distinct intracellular puncta, whereas stGnd1 was predominantly diffuse.

The additional requirement for autophagy and NVJ in the elimination of specific misfolded proteins, such as tGnd1, in quiescent cells suggests that clearance of these proteins by the UPS is inadequate. The availability of proteasomes does not appear to be the limiting factor, as clearance of another misfolded protein, stGnd1, did not require autophagy or NVJ. Rather, substrate-specific facilitators of UPS-mediated protein degradation, such as chaperones, may be limiting. Potential candidates for limiting factors include Sis1, a Hsp40 chaperone required for efficient degradation of tGnd1, but not stGnd1 in proliferating cells, and Hsp104, a protein disaggregase that translocates to the nucleus in quiescent cells.

In conclusion, our data indicate that quiescent cells maintain a pool of fully assembled proteasomes that remain actively engaged in degrading quality control substrates, while efficient clearance of specific misfolded proteins additionally requires the activity of autophagy and intact NVJs (Figure 1). The role of NVJs and autophagy in PQC becomes more prominent upon cell entry into quiescence. The basis of substrate selectivity and the molecular mechanisms underlying the involvement of autophagy and NVJs in the clearance of misfolded proteins in quiescent cells represent an interesting new avenue for future research. Notably, the accumulation of tGnd1 was particularly high in the deletion mutant of *VAC8*. Vac8 is a sole armadillo-repeat yeast protein that is involved in several pathways, including autophagy, NVJ formation and docking of lipid droplets to the vacuolar membrane. Future studies may reveal the relative contribution of each of these Vac8-dependent processes to PQC in quiescent cells and their potential interplay with UPS-mediated degradation. Finally, while this study focused mainly on early quiescence, it is possible that the dependence of PQC on specific pathways changes in the later phases of quiescence. Although data on PQC in quiescent cells of mammalian organisms are limited, existing evidence suggests that upregulation of autophagy may be a common feature. Since NVJ-associated proteins share functional similarities with membrane contact sites found in mammalian cells, these mechanisms may be evolutionarily conserved.

**Figure 1.**
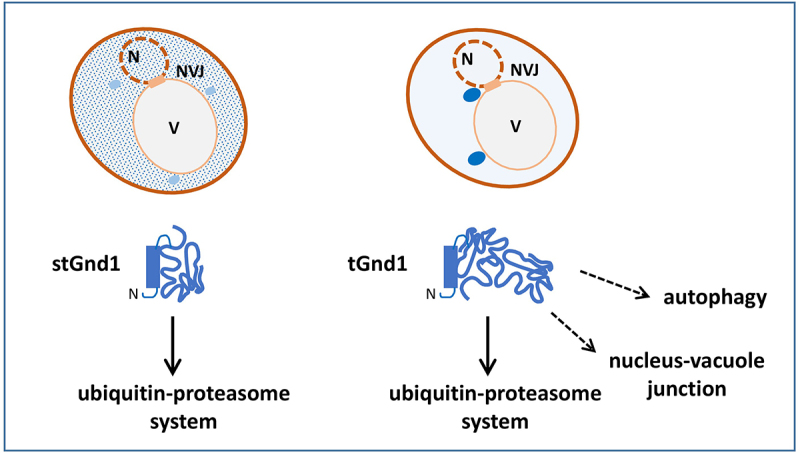
Quiescent yeast cells retain the ability to mitigate misfolded proteins by degradation-mediated protein quality control. Misfolded proteins tGnd1 and stGnd1 expressed in quiescent cells of yeast *Saccharomyces cerevisiae* are targeted to selective degradation by the UPS. Efficient clearance of tGnd1 in quiescent cells additionally depends on autophagy and NVJs. While the intracellular localization of stGnd1 is predominantly diffuse, tGnd1 forms distinct inclusions. N, cell nucleus; V, vacuole (yeast counterpart of the lysosome).

## Abbreviations

Cue5: Coupling of Ubiquitin conjugation to ER degradation;
Gnd1: 6-phosphogluconate dehydrogenase;
Hsp104: heat shock protein 104;
Hsp40: heat shock protein 40;
NVJ: nucleus-vacuole junction;
Nvj1: nucleus vacuole junction 1;
PQC: protein quality control;
Rpn11: Regulatory Particle Non-ATPase11;
San1: Sir Antagonist1;
Sis1: SIt4 Suppressor1;
Ubr1: Ubiquitin Protein Ligase E3 Component N-Recognin 1;
UPS: ubiquitin-proteasome system;
Vac8: VACuole related 1.

## References

[1] Franić D, Pravica M, Zubčić K, et al. Quiescent cells maintain active degradation-mediated protein quality control requiring proteasome, autophagy, and nucleus-vacuole junctions. J Biol Chem. 2025;301(1):108045. doi: 10.1016/j.jbc.2024.108045 39617269 PMC11731230

